# Vascular Function in Chronic Non-Communicable Diseases 2.0

**DOI:** 10.3390/biomedicines12122761

**Published:** 2024-12-04

**Authors:** Annalisa Noce, Manfredi Tesauro

**Affiliations:** 1 Department of Systems Medicine, University of Rome Tor Vergata, 00133 Rome, Italy; 2 UOSD Nephrology and Dialysis, Policlinico Tor Vergata, 00133 Rome, Italy

Chronic degenerative non-communicable diseases (CDNCDs) represent the main causes of mortality and morbidity worldwide [1,2]. Over the years, the number of individuals with CDNCDs has increased exponentially [3,4]. These data are related to unhealthy habits and to the increase in life expectancy [5], and this statement is predominantly evident in Countries with a higher percentage of elderly people [6,7].

Furthermore, CDNCDs in developed Countries significantly impact the National Health Expenditure [8]. Endothelial dysfunction contributes to the development of CDNCDs [9,10], since in this pathological conditions, vascular endothelial cells are characterized by a proinflammatory and a prothrombotic phenotype [11]. In this regard, oxidative stress and micro-inflammatory status are peculiar features of the CDNCDs [12].

Adjuvant therapies against vascular dysfunction include moderate physical exercise [13], healthy eating habits, and the use of drugs exerting endothelium-protective action [14,15,16].

In our Special Issue, we have published interesting papers within this field of research. In particular, Chen MC et al. [17] investigated the possible relationship between serum adiponectin levels and endothelial function in CKD patients under conservative therapy, since it is known that CKD contributes to the development of endothelial impairment [18]. The authors highlighted a direct correlation between adiponectin levels and the vascular reactive index.

Since it has been found that perivascular adipose tissue appears to be involved in endothelial dysfunction pathogenesis [19], it could contribute to atherosclerosis evolution and to insulin resistance. In an innovative review, Valentini A. et al. analyzed its role on cardiovascular diseases and type 2 diabetes mellitus onset, examining at the same time possible therapeutic treatments [20].

In a model of obese mice, Schreier B. et al. [21] studied the function of vascular smooth muscle cells (VSMCs) and endothelial cells (ECs) of epidermal growth factor receptors (EGFRs) in blood pressure regulation and in acute vascular reactivity. These authors pointed out that EGFRs, mainly those in VSMCs and, to a lesser extent, those in ECs, regulate short-term vascular reactivity, induced by angiotensin II, catecholamine, and volume load, both in lean and obese mice.

Additionally, in the field of endothelial dysfunction, Janina Krug et al. [22] evaluated the levels of soluble endothelial protein C receptors sEPCRs in the plasma of patients with peripheral artery disease (PAD), and they compared these levels with protein C activity and the biomarkers of endothelial function, inflammation, and angiogenesis. The authors suggested that circulating levels of sEPCRs may represent an important biomarker of endothelial dysfunction, including angiogenesis, and demonstrated that the progressive loss of endothelial protein C receptors may play a role in the development and the progression of PAD.

Myocardial ischemia/reperfusion injury (MIRI) is a pathophysiological process connected to the onset of numerous heart disorders. In their elegant review, Sagris M et al. discussed [23] the main innovations in the field of MIRI research, in particular, the recognition of associated mechanisms and the identification of potential new targets for therapeutic interventions. The authors conclude by reiterating the important role of MIRI in clinical practice, especially in interventional cardiology. Among the different pathophysiological mechanisms, the role of the endothelium has been emphasized. They conclude that despite extensive efforts in clinical and laboratory studies, there are currently no agents with a routine clinical role for MIRI prevention.

We conclude this analysis of the Special Issue with an interesting exploratory study on a rare clinical condition, Erythromelalgia (EM), also known as Mitchell’s disease, manifested by a clinical picture characterized by acute burning pain, erythema, and discomfort, especially in the extremities [24]. This pathology, which little is known, represents a great challenge from both a diagnostic and therapeutic point of view. The authors measured the perfusion responses of an EM patient to suprasystolic pressure (post-occlusive reactive hyperemia, PORH) and to controlled cold temperature, then they compared the obtained data with those of a healthy subjects group. All responses were registered with laser Doppler flowmeter (LDF) technology and then “decomposed” with Wavelet transform (WT). The results of the study, despite obvious limitations, showed significant deviations in the values of physiological components derived from the WT compared to those of the healthy subjects. The authors conclude that the agreement reached with the clinical evaluation provides the opportunity to use this approach in other patients with vascular diseases.
